# Synaptic vesicle pools are a major hidden resting metabolic burden of nerve terminals

**DOI:** 10.1126/sciadv.abi9027

**Published:** 2021-12-03

**Authors:** Camila Pulido, Timothy A. Ryan

**Affiliations:** Department of Biochemistry, Weill Cornell Medical College, New York, NY 10065, USA.

## Abstract

The brain is a metabolically fragile organ as compromises in fuel availability rapidly degrade cognitive function. Nerve terminals are likely loci of this vulnerability as they do not store sufficient ATP molecules, needing to synthesize them during activity or suffer acute degradation in performance. The ability of on-demand ATP synthesis to satisfy activity-driven ATP hydrolysis will depend additionally on the magnitude of local resting metabolic processes. We show here that synaptic vesicle (SV) pools are a major source of presynaptic basal energy consumption. This basal metabolic processes arises from SV-resident V-ATPases compensating for a hidden resting H^+^ efflux from the SV lumen. We show that this steady-state H^+^ efflux (i) is mediated by vesicular neurotransmitter transporters, (ii) is independent of the SV cycle, (iii) accounts for up to 44% of the resting synaptic energy consumption, and (iv) contributes substantially to nerve terminal intolerance of fuel deprivation.

## Teaser

The cost of not thinking: unused synapses are metabolically expensive due to persistent activity of a synaptic vesicle proton pump.

## INTRODUCTION

The human brain generally has a very small safety factor with respect to fuel supply, such that when blood glucose levels drop by only ~2-fold, severe neurological consequences ensue. As a result, even brief interruptions in blood flow that restrict delivery of glucose and oxygen, key elements necessary for adenosine 5′-triphosphate (ATP) synthesis, rapidly lead to severe neurological impairment. We previously demonstrated that nerve terminals are the likely loci of this metabolic vulnerability as they are remarkably sensitive to brief fuel supply interruption, showing complete cessation of synaptic vesicle recycling within minutes of fuel deprivation (*1*–*3*). We surmise that this vulnerability reflects the limits of the local ATP synthetic machinery’s ability to balance both increased ATP demand driven by activity and fundamental basal metabolic costs that are always incurred, independent of synaptic drive. Relatively, little is known about basal metabolic costs, particularly in specialized cellular locations that operate distally from the cell body. As a whole organ, the brain is energetically expensive, estimated to consume ~8 to 10 times the amount of energy per weight compared to average tissue because it consumes ~20% of the body’s fuel intake but represents only ~2 to 2.5% of the mass. Although a large fraction of this energy consumption is driven by electrical activity, the brain also likely has high resting metabolic rates because severe curtailment of brain electrical activity only decreases fuel consumption two- to threefold (*4*–*6*). The presence of large resting metabolic rates likely impose notable constraints on how well different neuronal compartments can adapt to changing metabolic needs. Because basal metabolic needs constitute a constant drain on resources, they will, in part, determine the ability of a given compartment to withstand temporary limitations in fuel supply, because any locally produced ATP is already “tapped” for handling resting needs. To determine whether nerve terminal metabolic vulnerability might be driven by local basal metabolic rates, we designed experiments to characterize this parameter and to uncover its molecular underpinning. Here, we show that nerve terminals have a high resting metabolic energy demand, independent of electrical activity, and that synaptic vesicle (SV) pools are a major source of basal energy consumption in this compartment, while the plasma membrane Na^+^/K^+^-ATPase (Na^+^- and K^+^-dependent ATPase) is only a minor contributor. We demonstrate that this basal metabolism arises from SV-resident vacuolar-type ATPase (V-ATPases) compensating for a previously unknown constant H^+^ efflux from the SV lumen. We show (i) that this steady-state H^+^ efflux is mediated by the vesicular neurotransmitter transporter, independent of the SV cycle; (ii) that it accounts for about half of the resting synaptic energy consumption; and (iii) that suppression of this transporter activity significantly improves the ability of nerve terminals to withstand fuel withdrawal. Our findings underscore why nerve terminals are susceptible to metabolic compromises, as in addition to regulating ATP production in responses to activity, they must constantly meet a large local metabolic burden.

## RESULTS

### The vesicular proton pump, not the plasma membrane Na^+^/K^+^-ATPase, accounts for a large resting basal ATP consumption in nerve terminals

**Fig. 1.**
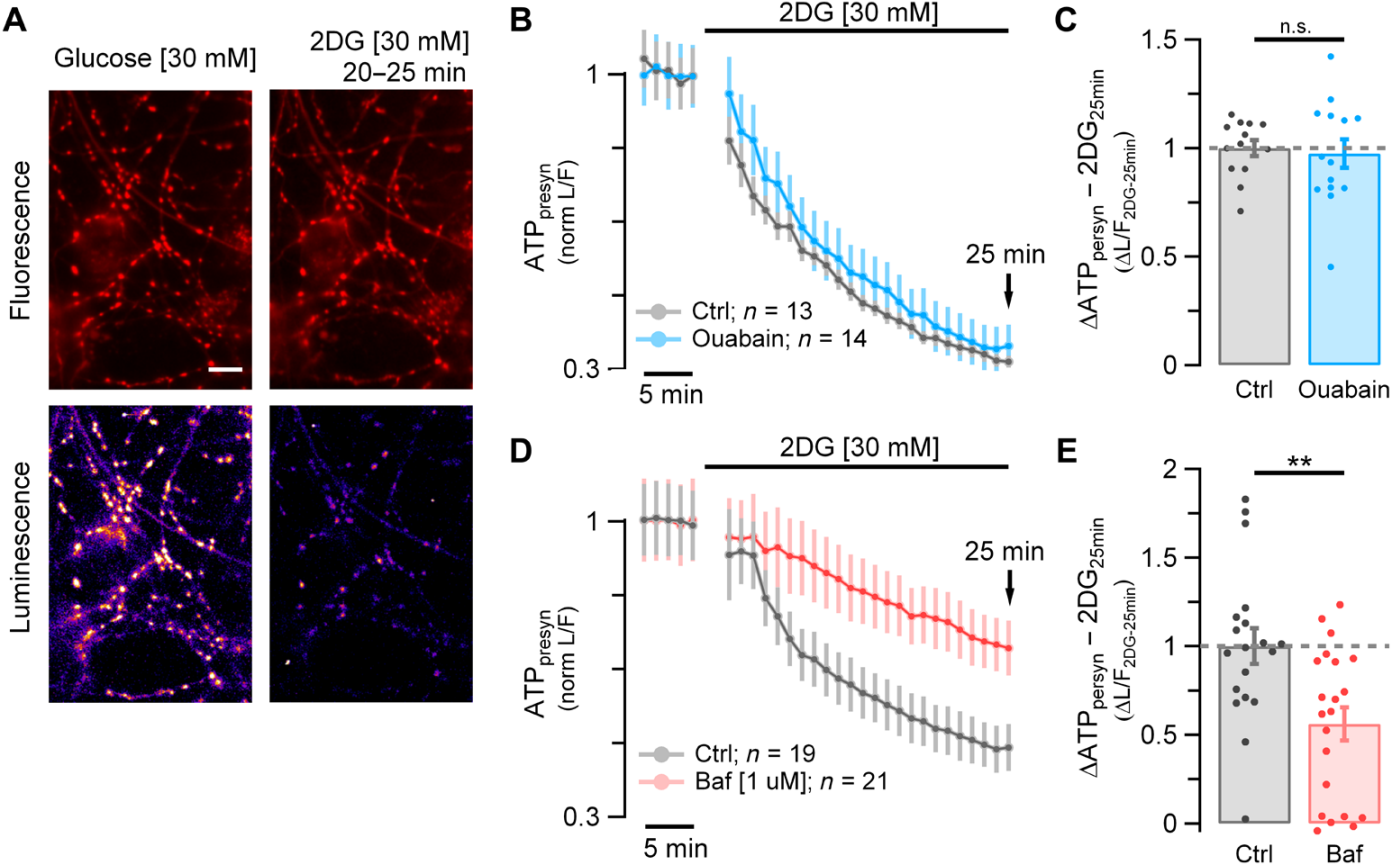
SV V-ATPase, not the plasma membrane Na^+^/K^+^-ATPase, is a primary energy burden in resting synapses. (**A**) Syn-ATP fluorescence (F) (top) and luminescence (L) (bottom) images acquired from primary hippocampal neurons in glucose (left) and 25 min after replacing glucose with 2DG (right) in the presence of TTX (five frames average taken at 20 to 25 min). Scale bar, 10 μm. (**B** and **D**) Average ATP_presyn_ (L/F intensity ratio) time course normalized to baseline measured in glucose. (B) Ensemble average time course from neurons incubated in 2DG (*n* = 13; gray trace) or incubated in 2DG + ouabain [1 mM] (*n* = 14; blue trace). (**C**) Average of ΔATP_presyn_ (ΔL/F) values after 25 min in 2DG (2DG_25min_) in the presence of ouabain (blue dots) normalized to control (gray dots): mean ± SEM: 0.97 ± 0.065 versus 1 ± 0.037. (D) Ensemble average time course from neurons incubated in 2DG (*n* = 19; gray trace) or incubated in 2DG + bafilomycin [1 μM] (*n* = 21; red trace). (**E**) Average ΔATP_presyn_ (ΔL/F) values after 25 min in 2DG (2DG_25min_) in the presence of bafilomycin (red dots) normalized to control (gray dots): mean ± SEM: 0.56 ± 0.093 versus 1 ± 0.1. Error bars indicate SEM. **P < 0.01, Wilcoxon-Mann-Whitney test. n.s., not statistically significant.

We developed a quantitative approach to determine the magnitude of the resting metabolic load at synapses. We measured the kinetics of presynaptic ATP (ATP_presyn_) decline in resting synapses of dissociated hippocampal neurons after acute fuel deprivation using a quantitative genetically encoded optical reporter, Syn-ATP (Fig. 1A) (*1*). In the presence of the Na^+^ channel blocker tetrodotoxin (TTX), replacing glucose with the nonmetabolizable 2-deoxyglucose (2DG) led to a rapid continuous depletion in ATP_presyn_ down to 32% ± 1.4 (*n* = 13) of the original ATP_presyn_ levels in 25 min (Fig. 1B, gray) with an initial slope of ~5.4%/min. In general, the resting ATP concentration in most cells is expected to be in the millimolar range. Consistent with this, we previously showed that under resting conditions ATP_presyn_ is ~1.4 mM, which allows to provide an estimate of the total number of ATP molecules being consumed per unit time of ~3100 ATP/s per nerve terminal (see Methods). The action of the Na^+^/K^+^-ATPase to restore ionic gradients is considered to be one of the largest metabolic costs in the active brain (*7*–*9*). In the absence of action potencial (AP) firing, the Na^+^/K^+^-ATPase compensates for any leak currents across the plasma membrane. We sought to determine the extent to which this enzyme activity might contribute to the resting metabolic load by examining the kinetics of ATP depletion during fuel deprivation as above but in the presence of ouabain, a potent inhibitor of the Na^+^/K^+^-ATPase pump. Although ouabain application (1 mM) in the absence of TTX drives axonal membrane depolarization in our system (fig. S1, A and B), unexpectedly, inhibiting the Na^+^/K^+^-ATPase had no measurable impact on the kinetics of ATP depletion (Fig. 1B, blue): In the presence of 1 mM ouabain and TTX, after 25 min of 2DG incubation, ATP_presyn_ decreased to very similar levels as controls [normalized depletion mean ± SEM; Ctrl: 1 ± 0.037 (*n* = 13) versus ouabain: 0.97 ± 0.065 (*n* = 14); n.s., *P* = 0.98; Fig. 1C]. These data suggest that relatively few Na^+^ and K^+^ ions need to be pumped across the plasma membrane to maintain the resting membrane potential. As the experiments are done in the presence of TTX, any depolarization will not lead to aberrant AP firing; however, it is less clear what the impact of a slow depolarization of the membrane potential will be on the activity of axonal voltage-gated Ca^2+^ channels over the time course of ATP depletion. As Ca^2+^ influx itself can trigger numerous biological processes that might, in turn, confound interpretations of metabolic burdens, we carried out measurements of resting Ca^2+^ levels following ouabain application in TTX (fig. S1, C and D). These experiments showed that in the presence of TTX and ouabain, resting Ca^2+^ levels remained at baseline for >30 min. We surmise from these experiments that although the Na^+^/K^+^-ATPase is active in axons under resting conditions, it imposes only a low ATP hydrolysis rate and is not a significant contributor to the resting metabolic load at nerve terminals.

We therefore considered alternative mechanisms at presynaptic terminals to explain basal metabolic dynamics. SVs use the proton motive force generated by vesicle-resident V-ATPases, with one to two copies per vesicle (*10*), each hydrolyzing three ATP molecules per 10 protons translocated (*11*) to power neurotransmitter uptake into the vesicle lumen (*12*). Although one expects ATP expenditure for filling vesicles with neurotransmitter, it is less clear in a resting vesicle pool whether there is any residual activity that might account for part of the resting metabolic load, a state when one expects most SVs to be full of neurotransmitter. We tested for this possibility by examining basal ATP consumption (in TTX and 2DG) in the presence of bafilomycin, a potent V-ATPase inhibitor (*13*). These experiments showed that, unlike with blockade of the plasma membrane Na^+^/K^+^ pump, blocking V-ATPases significantly reduced basal ATP consumption. The initial ATP_presyn_ depletion rate in the presence of 1 μM bafilomycin was ~3 times slower that in control [incubation time at which the ATP_presyn_ content drops by 25%, *t*_1/4-baf_ = 22.01 min ± 3.6 (*n* = 21), *t*_1/4-ctrl_ = 7.69 min ± 1.36 (*n* = 19); ****P < 0.0001; Fig. 1C], leaving ~69.91% ± 5.0 of the original ATP at 25 min compared to just 46.41% ± 5.35 in controls (**P < 0.01; Fig. 1D). The ATP values normalized to Ctrl_25min_ are, respectively, 0.56 ± 0.093 versus 1 ± 0.1 (**P < 0.01; Fig. 1E). These data unmask the V-ATPase as a main energy burden in resting synapses that accounts slightly less than half of the resting ATP consumption. Given the large total number of nerve terminals in the brain and that this burden will be present constantly, these results imply that the resting SV pools in total likely constitute a substantial energy burden in the brain.

### A “hidden” H^+^ flux from the SV lumen is present even in the absence of SV exocytosis and recycling

**Fig. 2.**
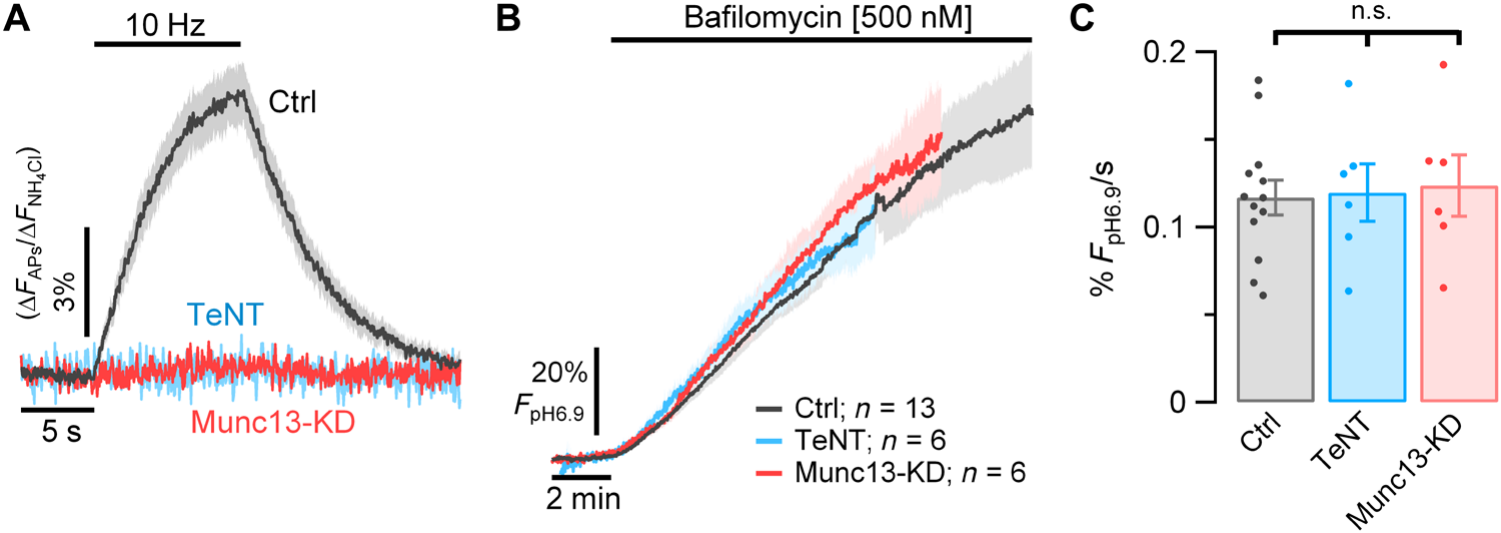
Resting SVs have a constant H^+^ efflux. H^+^ flux from SVs measured in hippocampal neurons expressing vG-pH. (**A**) Average vG-pH traces in response to 100 APs (10 Hz) in control neurons (Ctrl, gray trace, *n* = 13) or in neurons where exocytosis is genetically suppressed by either ablating expression of Munc13 (Munc13-KD, red trace, *n* = 6) or expressing TeNT (blue trace, *n* = 6). Δ*F* values are normalized to maximal Δ*F* from NH_4_Cl treatment (Δ*F*/Δ*F*_NH4Cl_). (**B**) vG-pH average traces of Ctrl, Munc13-KD and TeNT neurons measured in the presence of TTX before and after perfusion with bafilomycin. Fluorescence expressed as a percentage of the fluorescence expected for pH 6.9 (% *F*_pH6.9_) based on perfusion with NH_4_Cl (see Methods). ±SEM intervals are indicated by shaded colored areas. (**C**) Bafilomycin application causes SV vG-pH fluorescence to increase at the same rate in all three conditions and thus is not related to exocytosis. Average rates of alkalization for Ctrl, Munc13-KD, and TeNT neurons measured over the first 6 min in bafilomycin, respectively, as 0.117% *F*_pH6.9_/s ± 0.01, 0.124% *F*_pH6.9_/s ± 0.018, and 0.119% *F*_pH6.9_/s ± 0.016.

V-ATPases are electrogenic pumps and as such only stop burning ATP when the energy barrier for moving a proton against a chemical potential exceeds the energy provided by hydrolyzing ATP. Our results imply that the H^+^ gradient in SVs is constantly dissipated but restored by the V-ATPase, which when blocked would lead to alkalization of SVs. To test this hypothesis, we measured changes in SV luminal pH (pH_Lum_) from resting nerve terminals of neurons expressing a vesicular glutamate transporter (vGlut) tagged with the pH sensor pHluorin, vGlut-pHluorin (vG-pH) before and after blocking the V-ATPase with bafilomycin, a macrolide that binds to and inhibits the cytoplasmic ATPase rotor (*13*). Before blocking the proton pump, the basal fluorescence of boutons expressing vG-pH remained stable over time (2 min baseline measurements, Fig. 2B). However, upon addition of bafilomycin, the vG-pH fluorescence immediately began to increase at a rate of 0.117 ± 0.01% *F*_max_/s (*n* = 13), where *F*_max_ is the fluorescence at pH of 6.9, the pH of the cytoplasm, referred to herein as *F*_pH6.9_ (Fig. 2, B and C, black trace). These data indicate that there is a steady-state efflux of H^+^ from SVs, which, in turn, is being compensated by the action of the V-ATPase, at the expense of ATP hydrolysis. This H^+^ efflux showed a reasonably strong temperature dependence, as lowering the temperature from 37° to 25°C reduced the rate by a factor of ~2.1 (fig. S2). Similar results were obtained using an alternate V-ATPase blocker, folimycin (fig. S3), with only minor differences between the potency of the two pump blockers, which saturate near ~500 nM. These data imply that the flux of H^+^ is occurring from resting SVs; however, these changes in pH would also occur if the SVs underwent spontaneous exocytosis, releasing protons to the extracellular space and retaining an alkaline lumen when recycling in the presence of bafilmocyin. To determine the possible contribution of this mechanism to our measurements, we carried out two types of experiments. The first was to repeat the H^+^ flux measurements in synapses where we strongly suppressed exocytic mechanisms by either genetically expressing tetanus-toxin light chain (TeNT), which cleaves the major vesicle-associated protein required for synaptic exocytosis, vesicle-associated membrane protein isoform 2 (*14*, *15*), or by suppressing expression of Munc13-1/2 using a short hairpin RNA (shRNA) targeting the genes encoding these proteins (*16*). In hippocampal neurons, a combination of Munc13-1 and Munc13-2 is required for all known forms of SV exocytosis (*17*). Suppressing Munc13-1/2 expression eliminates both AP potential evoked exocytosis (Fig. 2A) and hypertonic sucrose-driven asynchronous vesicle fusion (fig. S4). Suppressing exocytosis with either of these approaches had no quantitative impact on the magnitude of the H^+^ flux from the resting vesicle pool: Ctrl: 0.117% *F*_pH6.9_/s ± 0.01 versus Munc13-Knockdown (KD): 0.124% *F*_pH6.9_/s ± 0.018 (*n* = 6; n.s., *P* = 0.7) and TeNT: 0.119% *F*_pH6.9_/s ± 0.016 (*n* = 6; n.s., *P* = 0.9; Fig. 2, B and C). These experiments show conclusively that the fluorescence increase following proton pump block occurs without any exocytosis.

**Fig. 3.**
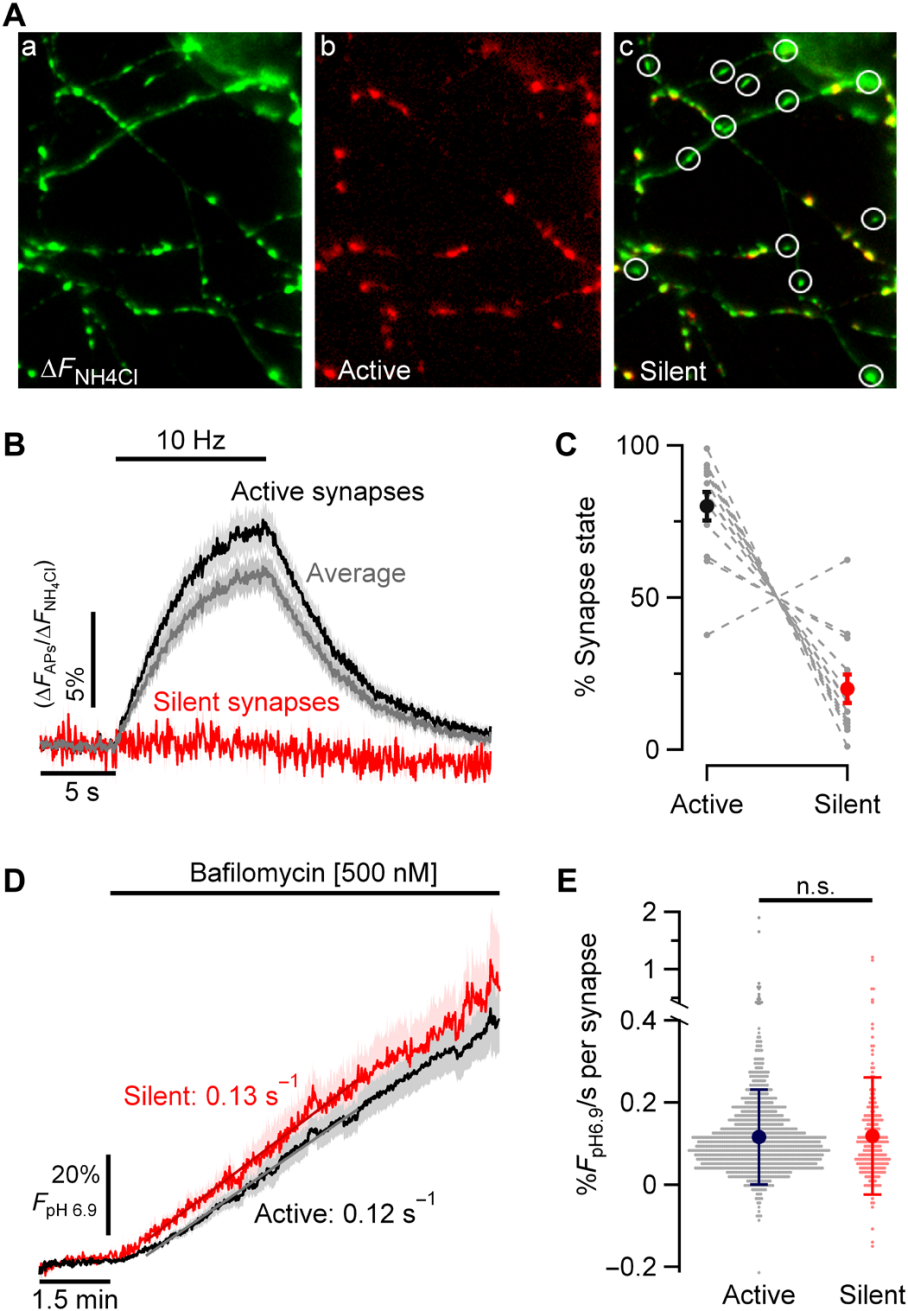
SV H^+^ efflux is not correlated with individual synapse release properties. (**A**) Representative images showing variation in synaptic release in an individual neuron expressing vG-pH. Perfusion with NH_4_Cl perfusion [(**a**) pseudo-colored green]reveals expression pattern of vG-pH across synapses. vG-pH responses to electrical activity [100 APs (10 Hz); (**b**) pseudo-colored red] shows that a portion of the nerve terminals are silent [(**c**) overlay between left and middle images] indicated by circles. (**B**) Average vG-pH response to stimulation from active (black), silent (red), and total synapses (gray) per neuron (*n* = 13); where silent synapse is defined as showing responses less than 1.2 SD of the prestimulus baseline. (**C**) Percentage of nerve terminals in each category on a neuron-by-neuron basis (gray dashed lines) shows that ~80% of nerve terminals show robust responses and 20% are silent. (**D**) Ensemble average of individual neuron responses of SV H^+^ efflux kinetics measured in TTX and bafilomycin for active and silent synapses across 13 neurons (active: 0.12% *F*_pH6.9_/s ± 0.013 and silent: 0.13% *F*_pH6.9_/s ± 0.021; *P* = 0.24). (**E**) Show that the SV H^+^ efflux is unrelated to individual bouton exocytic properties [active synapses: mean ± SD: 0.12% *F*_pH6.9_/s ± 0.12 (*n* = 1035) and silent synapses: 0.12% *F*_pH6.9_/s ± 0.14 (*n* = 239)].

A second approach was to examine the apparent H^+^ flux in synapses that show no evoked synaptic responses even in their native state. Previously, we showed that for any given axon, a small portion (~15 to 20%) of synaptic boutons show no evoked exocytosis in response to AP bursts and are intermixed randomly with secretion-competent boutons in the same axon (*18*). We first measured the size of action potential–evoked signals following a 100 APs (10 Hz) stimulus train across all boutons using vG-pH normalized to the maximal signal obtained during NH_4_Cl perfusion (Fig. 3, Aa and B). We defined a “silent” synapse as one that failed to show an AP-evoked change greater than 1.2 SD of the prestimulus baseline (Fig. 3Ac) and “active” synapses as those whose response cross this threshold (Fig. 3 Ab). Using these criteria, 80% ± 4.7 (*n* = 13) of synaptic boutons in our experiments were scored as active (Fig. 3C), consistent with previous findings (*19*, *20*). Subsequently, we carried out proton-pump blocking experiments in the same neurons in TTX. We then compared the H^+^ efflux rates in active and silent synapses (Fig. 3D). These experiments showed that H^+^ efflux rates from individual boutons were unrelated to synaptic state defined as above [active synapses: mean ± SD: 0.12% *F*_pH6.9_/s ± 0.12 (*n* = 1035) and silent synapses: 0.12% *F*_pH6.9_/s ± 0.14 (*n* = 239); *P* = 1; Fig. 3E], solidifying the notion that resting SV pools have substantial sustained V-ATPase activity at the expense of ATP consumption.

### vGlut mediates the steady-state H^+^ flux from SVs

**Fig. 4.**
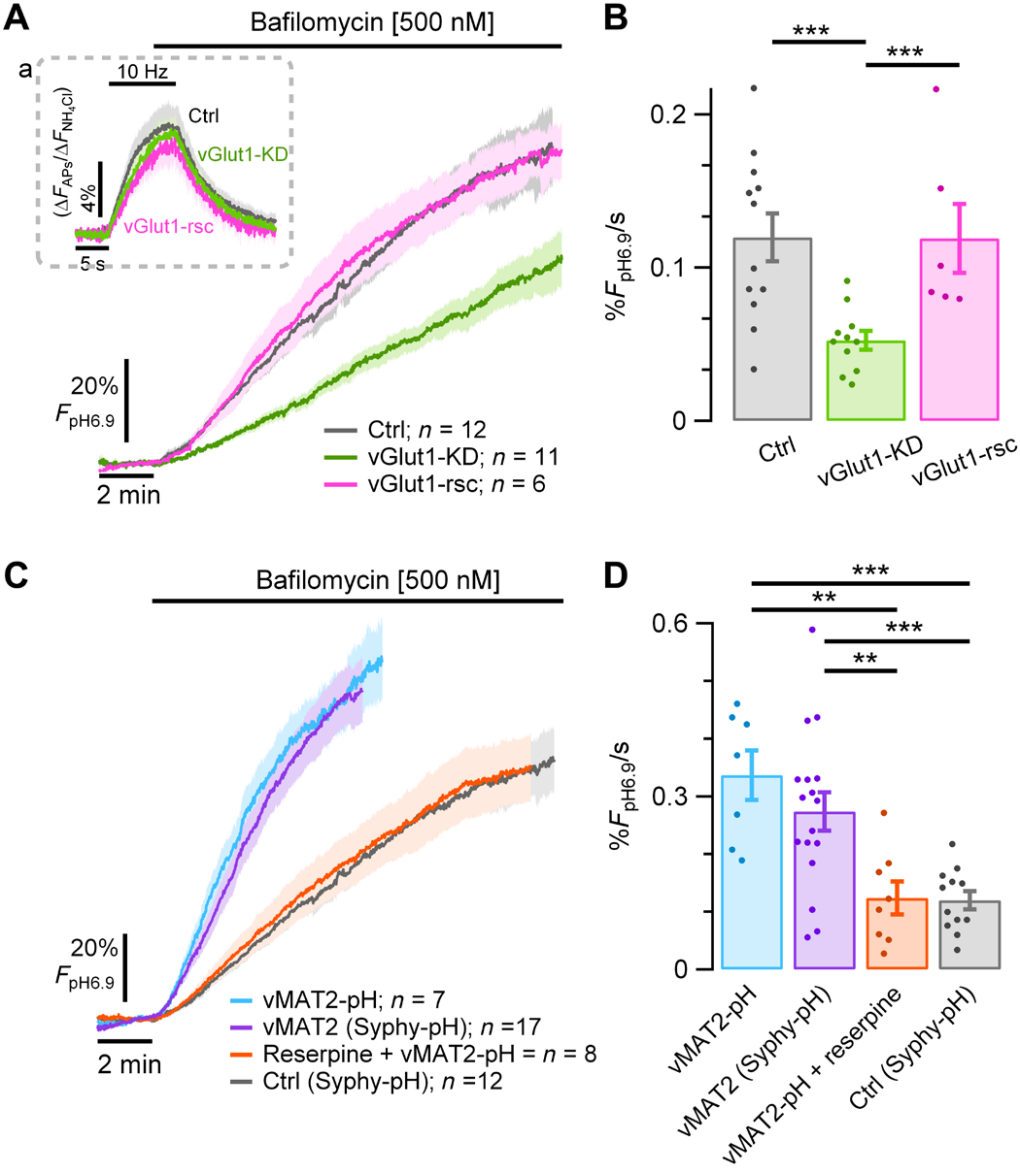
Vesicular neurotransmitter transporters (i.e., vGlut1 and vMAT2) mediate a large fraction of the resting SV H^+^ efflux. (**A** and **B**) SV H^+^ efflux was measured in the presence of TTX and bafilomycin in hippocampal neurons expressing either Syphy-pH (control, gray trace), Syphy-pH and an shRNA suppressing expression of vGlut1 (vGlut1-KD, green trace) or Syphy-pH, vGlut1-KD and a human variant of vGlut1, resistant to the ShRNA (vGlut1-rsc, pink trace). (A) Control, vGlut1-KD, and vGlut1-rsc show similar exocytic responses to electrical stimulation (inset), but vGlut1-KD neurons show much lower SV H^+^ efflux. (B) SV H^+^ efflux rates in Ctrl: 0.12% *F*_pH6.9_/s ± 0.016 (*n* = 12) versus vGlut1-KD: 0.052% *F*_pH6.9_/s ± 0.006 (*n* = 11), ***P < 0.001. SV H^+^ efflux is fully recovered in neurons where vGlut1 was rescued [0.12% *F*_pH6.9_/s ± 0.022 (*n* = 6) versus vGlut1-KD ***P < 0.001]. (**C**) Hippocampal neuronal expression of the exogenous transporter vMAT2, either coupled to pHluorin (vMAT2-pH, blue trace, *n* = 7) or coexpressed with Syphy-pH (vMAT2, violet trace, *n* = 17) led to a faster SV H^+^ efflux in the presence of bafilomycin, compared with control neurons expressing native vGlut1 (Syphy-pH, gray trace, *n* = 12). This increase in H^+^ flux is completely abolished by application of 100 nM reserpine (red trace, *n* = 8). (**D**) Bar plot of the SV H^+^ efflux rates of vMAT2-pH (0.34% *F*_pH6.9_/s ± 0.043), vMAT2 (0.28% *F*_pH6.9_/s ± 0.033), reserpine (0.124% *F*_pH6.9_/s ± 0.029) and control (0.12% *F*_pH6.9_/s ± 0.016). vMAT2-pH versus: Ctrl, ***P < 0.001 and reserpine, **P < 0.01. vMAT2 versus: Ctrl, ***P < 0.001 and reserpine, **P < 0.01. Error bars indicate SEM. **P < 0.01 and ***P < 0.001, Wilcoxon-Mann-Whitney test.

We next turned to identifying the molecular basis of the SV H^+^ efflux. All SV types use vesicular neurotransmitter transporters driven in part by the energy provided by the V-ATPase to drive the filling process via an alternating access mechanism (*21*). Although some vesicular neurotransmitter transporters are known to exchange H^+^ with neurotransmitter during the pumping process, it has been difficult to ascertain this for vGlut family members (*22*). We wondered whether vGlut might mediate the proton “leak.” To test this hypothesis, we used shRNA-mediated knockdown of vGlut1 expression (*23*) in neurons expressing a pHluorin-tagged synaptophysin (Syphy-pH). Consistent with our previous findings (*23*), loss of vGlut1 did not impair exocytosis (Fig. 4 Aa). Measurements of H^+^ efflux (in bafilomycin) from the SV pool in control neurons expressing Syphy-pH were similar to those measured with vG-pH (0.12% *F*_pH6.9_/s ± 0.016, *n* = 12; Figs. 2C and 4B), consistent with previous findings that vG-pH expression does not increase the copy number of vGlut1 at SV (*24*). However, knockdown of vGlut1 significantly reduced the H^+^ efflux rate [vGlut1-KD: 0.053% *F*_pH6.9_/s ± 0.006 (*n* = 11), ***P < 0.001; Fig. 4, A and B], without changing the initial SV pH. This reduction in SV H^+^ efflux in vGlut1-KD neurons was fully reversed upon expression of the human variant of vGlut1, which was resistant to the shRNA used for rat [vGlut1-rsc: 0.12% *F*_pH6.9_/s ± 0.022 (*n* = 6), *P* = 0.9; Fig. 4, A and B]. Quantitative immunofluorescence analysis showed that under these conditions, vGlut1 expression is reduced by ~80% (Fig. S5), somewhat lower than our previous estimate (*23*). Therefore, it is possible that the remaining H^+^ efflux following knockdown arises from residual vGlut1. This transporter is thus necessary for H^+^ efflux in resting SVs.

**Fig. 5.**
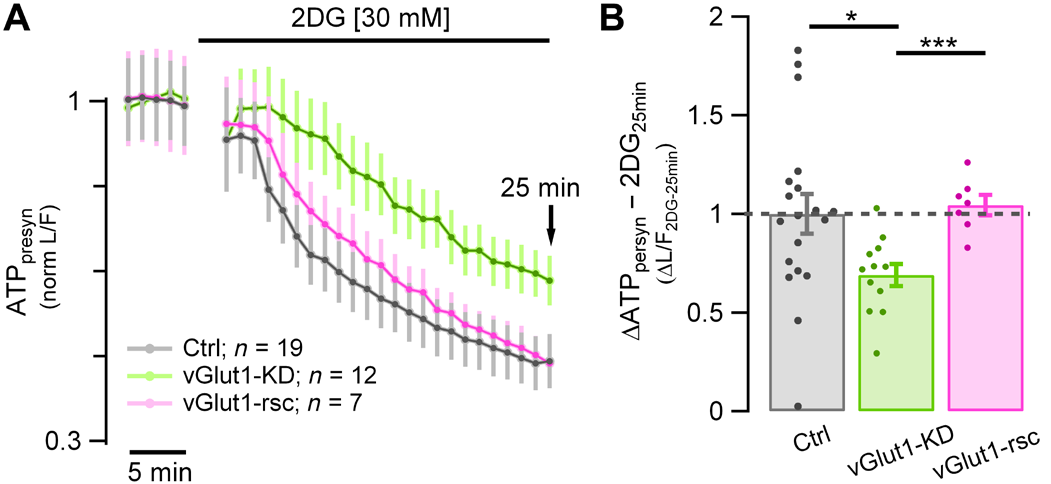
SV H^+^ efflux mediated by vGlut1 is compensated by the action of the v-ATPase. (**A**) Resting ATP_presyn_ consumption measured using Syn-ATP in TTX and 2DG expressed as normalized L/F in vGlut1-KD (green trace; *n* = 12), vGlut1-KD + vGlut1-rsc (pink trace; *n* = 7), and control neurons (gray trace; *n* = 19). (**B**) Suppression of vGlut1 expression lowers the resting ATP consumption by 33% (measured after 25 min in 2DG, 2DG_25min_) in vGlut1-KD neurons (green dots) normalized to control neurons (gray dots): mean ± SEM: 0.69 ± 0.056 versus 1 ± 0.1 (*P < 0.02). Resting ATP_presyn_ consumption is fully rescued compared to Ctrl levels (vGlut1-rsc: mean ± SEM: 1.05 ± 0.052, *P* = 0.6). Error bars indicate SEM. *P < 0.02 and ***P < 0.001, Wilcoxon-Mann-Whitney test.

### Exogenously expressed vMAT2 drives a reversible SV H^+^ efflux in hippocampal neurons

Although our data show that vGlut is necessary for a large fraction of the resting H^+^ flux from SVs, whether vGlut is a direct or indirect mediator of H^+^ flux is not clear from our experiments. It is possible that other proteins responsible for maintaining SV ionic flux balances during glutamate uptake are responsible for the H^+^ current. To address this problem, we sought to determine whether other vesicular neurotransmitter transporters, particularly ones considered to be bona fide proton exchangers, could mediate an H^+^ leak from SVs. We chose to measure H^+^ fluxes in neurons where we expressed an exogenous neurotransmitter transporter for which there is no cognate neurotransmitter molecule and which additionally can be blocked acutely using a pharmacological inhibitor. As hippocampal neurons do not express tyrosine hydroxylase (TH), they do not synthesize dopamine. Dopamine and serotonin are packaged into SVs in neurons that express TH by vesicular monoamine transporter (vMAT) family members. In addition, vMATs are known to exchange two protons for each monoamine taken up in the dopaminergic SV lumen (*25*, *26*). Expression of vMAT2-pHluorin (vMAT2-pH) (*27*, *28*) in hippocampal neurons was localized to nerve terminals with very low surface expression (not shown). Similar to our measurements with either vG-pH or Syphy-pH, application of bafilomycin led to a prompt increase in vMAT2-pH fluorescence. The rate of increase was ~2.8-fold greater than in nerve terminals expressing Syphy-pH [i.e., without vMAT2; vMAT2-pH: 0.34% *F*_pH6.9_/s ± 0.043 (*n* = 7) versus Syphy-pH: 0.12% *F*_pH6.9_/s ± 0.016 (*n* = 12); ***P < 0.001; Fig. 4, C and D]. As with vGlut1, the H^+^ flux was independent of tagging with pHluorin as expression of vMAT2 along with Syphy-pH showed very similar H^+^ flux rates compared to vMAT2-pH neurons (0.28% *F*_pH6.9_/s ± 0.033, *n* = 17; *P* = 0.35). However, application of reserpine, a potent inhibitor of vMAT2, completely suppressed the increase in H^+^ flux (0.124% *F*_pH6.9_/s ± 0.03, *n* = 8; **P < 0.01). These data, together with the vGlut1-KD experiments, strongly support the idea that the transporters themselves can mediate the SV H^+^ efflux.

### vGlut1-mediated SV H^+^ efflux accounts for up to ~44% of the high resting ATP consumption

Our data shows that a constant proton flux accounts for ~44% of resting synaptic ATP consumption (Fig. 1, D and E) and that this, in turn, arises because of a constant H^+^ efflux mediated by vGlut1. These data make a strong prediction that loss of vGlut1 should decrease resting ATP consumption, similar to simply blocking the V-ATPase. To test this hypothesis, we measured basal ATP consumption rates (in TTX and 2DG) in neurons where vGlut1 expression was suppressed. After removal of vGlut1, the ATP [expressed as luminescence to fluorescence ratio (L/F)] rate of decay in the presence of 2DG was slower than the control [*t*_1/4-vGlut1-KD_: 16.8 min ± 1.65 (*n* = 12) versus *t*_1/4-Ctrl_: 7.7 min ± 1.36 (*n* = 19); ***P < 0.001; Fig. 5A]; after 25 min of incubation, 63.03% ± 5.1 remained from the original ATP content, which is significantly higher than the 46.41% ± 5.6 left in control neurons (*P < 0.02). Figure 5B shows normalized ΔATP_presyn_ values of vGlut1-KD with respect to Ctrl_25min_ (0.69 ± 0.056 versus 1 ± 0.1, *P < 0.02). This reduction in the basal ATP consumption in vGlut1-KD neurons was fully recovered upon expression of vGlut1-rsc [values normalized to Ctrl_25min_: 1.044 ± 0.052 (*n* = 7), *P* = 0.61]. Furthermore, ATP_25min_ percentage in vGlut1-KD neurons was similar to the ones obtained with bafilomycin [values normalized to Ctrl_25min_: bafilomycin: 0.56 ± 0.093 (*n* = 21) versus vGlut1-KD: 0.69 ± 0. 056 (*n* = 12); *P* = 0.57; Fig. 1E versus Fig. 5B]. Although the relationship between vGlut1 copy number and V-ATPase activity is unknown, if we assume a linear relationship, this predicts that the vGlut1-KD would suppress ATP consumption by ~35% (0.8 × 44%, the latter being the reduction obtained with bafilomycin), only slightly higher our measured value (31%). Overall, the results reveal that the SV pool is a major source of metabolic activity even in the absence of electrical activity and neurotransmission.

### Suppressing vGlut1 expression sustains nerve terminal function in restricted fuel conditions

**Fig. 6.**
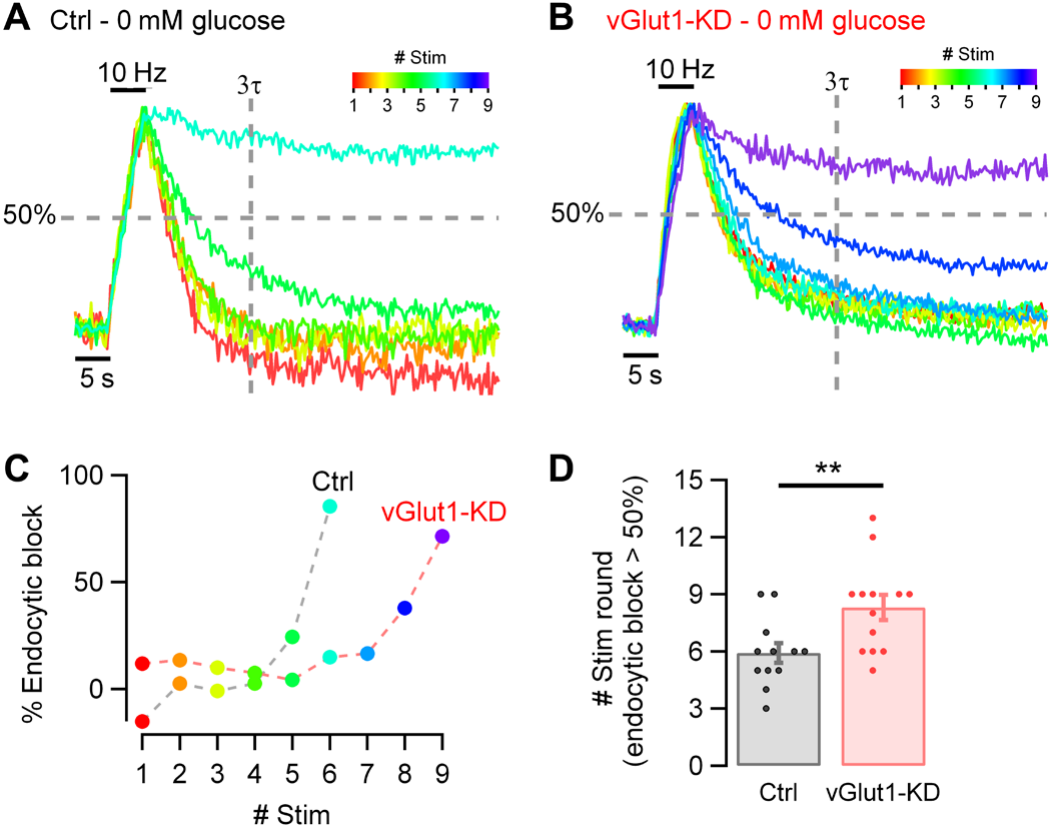
Basal SV energy consumption affects synaptic function in hypometabolic conditions. (**A** and **B**) Synaptic function measured in hippocampal neurons expressing Syphy-pH in the absence of glucose (0 mM glucose). Color-coded traces represent responses to one stimulus round (50 APs, 10 Hz) applied every minute (from red = first round to violet = ninth round). Responses are normalized to the peak. Vertical dashed line represents three times τ measured at the first round. Horizontal dashed line represents 50% retrieval of the exocytic signal. (A) Traces to six stimulus rounds in a control neuron. (B) Traces to nine stimulus rounds in a neuron suppressing expression of vGlut1 (vGlut1-KD). (**C**) Percentage of endocytic block from the examples shown in (A) and (B), measured each round at 3τ time point. Suppression of vGlut1 expression significantly prolongs the ability to recycle SVs in restricted fuel conditions. (**D**) Number of rounds of stimulation before endocytic block exceeds 50% is smaller in neurons expressing vGlut1 (gray dots, *n* = 12) than in vGlut1-KD neurons (red dots, *n* = 13): mean ± SEM: 5.92 rounds ± 0.52 versus 8.31 rounds ± 0.67. Error bars indicate SEM. **P < 0.01, Wilcoxon-Mann-Whitney test.

Our data imply that the H^+^ efflux from SVs contributes to a high resting metabolic rate. As one of the primary manifestations of a compromise in fuel availability at nerve terminals is an arrest in SV recycling, we speculated that suppressing vGlut1 expression would allow nerve terminals to sustain SV recycling for longer periods under hypometabolic conditions, as the total ATP burden would be lower. To test this idea, we used Syphy-pH expressed in hippocampal neurons to measure SV recycling kinetics in response to activity in sequential rounds of 50 APs (10 Hz) bursts delivered at 1 min intervals immediately after all glucose was removed in both control neurons and those in expressing an shRNA targeting vGlut1 (Fig. 6). To drive a hypometabolic state, we reduced the extracellular glucose concentration to zero. Under these conditions, all energetic needs must presumably be met by either residual ATP or intermediates in ATP production because no external combustible carbon source is being provided. In control neurons, the removal of glucose led to the gradual slowing of SV recycling, here shown color-coded with respect to stimulus round (Fig. 6A), with the onset of slowing apparent in 5 min and a complete arrest apparent at the sixth round of AP bursts (Fig. 6, A, C, and D), consistent with our previous findings (*1*, *2*). To express the impact of SV recycling quantitatively, we measured the fraction of the exocytic signal that remained after defined poststimulus time period (3 × the decay time constant in the first round, 3τ), which we term the percentage of endocytic block (= ~5% in the first round by definition for a perfect exponential decay). For a given neuron, we determined the number of stimulus rounds it took for SV recycling to fail to retrieve 50% of the exocytic signal at this time period, which in control neurons was, on average, 5.9 rounds ± 0.5 (*n* = 12; Fig. 6D). In contrast when vGlut1 expression is suppressed, in the absence of any fuel, nerve terminals can now sustain function for ~50% more activity [8.31 rounds ± 0.67 (*n* = 13)] of recycling before SV recycling arrests (**P < 0.01; Fig. 6, B to D). These experiments demonstrate that the energetic burden created by the H^+^ efflux mediated by vGlut1, strongly affects the efficacy of the SV cycle when fuel availability is restricted.

## DISCUSSION

Our studies provide a compelling explanation for the reason nerve terminals are so sensitive to metabolic compromise and, in turn, potentially speak to why brain tissue, in general, has a resting metabolic rate that is much higher than other tissues. The presence of SVs themselves and their intrinsic reliance on the V-ATPase to power neurotransmitter filling have rendered them sensitive to routes of efflux for H^+^ from the vesicle lumen, which, in turn, sustains V-ATPase activity, creating a constant energetic burden. Our experiments do not pin down the precise route of H^+^ efflux in glutamatergic vesicles but do show that vGlut is a critical mediator. Previous experiments (*12*) provided compelling evidence that luminal protons allosterically modulate glutamate transport by vGluts, an idea bolstered by recent ultrastructural studies of this transporter (*29*). The recent structural insights argue for an exchange for Cl^−^ ions with glutamate during the transport process, which is facilitated by low luminal SV pH. In this model, the pH gradient created by V-ATPases is only dissipated during exocytosis, not by the transport process. Our data indicate that even in the absence of exocytosis, SVs dissipate the H^+^ gradient over a ~10-min period in a manner that clearly depends on vGlut. This dissipation occurs in SV vesicles that have almost certainly reached a steady state with respect to neurotransmitter filling, as it occurs in synapses that have either very low release probability (Fig. 3) or in which exocytosis has been prevented genetically (Fig. 2). Thus, it is unlikely that the H^+^ efflux requires active transport of glutamate into the vesicle. Expression of vMAT2 in a neuron that lacks monoamines also resulted in H^+^ efflux that was acutely prevented by a blocker of the transport mechanism. These data support the idea that the transporters themselves likely directly mediate H^+^ efflux but without neurotransmitter transport. A parsimonious explanation is that the energy barrier for switching between conformations in the vesicular neurotransmitter transporters can be driven at a low rate by thermal fluctuations, even in the absence of cargo (neurotransmitter) binding on the cytosolic facing conformation. If this occurs, and if protons are normally exchanged for neurotransmitter as part of the alternate access model, a proton would then get ejected from the SV vesicle lumen, essentially driven purely by thermal fluctuations.

The much higher H^+^ efflux generated by expressing vMAT2 in hippocampal neurons suggests that the thermal activation of the transport process is larger for this transporter than for vGlut (and therefore the energy barrier for the relevant conformational transition is lower) (Fig. 4). Part of this might arise from a higher stoichiometry of H^+^/dopamine exchange compared to a putative H^+^/glutamate exchange or a higher copy number of expressed vMAT2 transporters in our experiments. The latter seems unlikely because any vMAT2 expression is in addition to the resident vGlut transporters. One could therefore predict that in dopaminergic terminals, where vMAT2 is natively expressed, the resting SV H^+^ efflux would be much larger. This, in turn, predicts an increased energetic burden at rest, perhaps making dopaminergic neurons inherently more susceptible to metabolic compromise. It will be interesting in the future to see if the H^+^ efflux is a general property of all vesicular neurotransmitter transporters, beyond vMAT and vGlut. GABAergic SVs appear to have very different reacidification dynamics and resting pH compared to glutamatergic vesicles, both of which are dependent on the vesicular γ-aminobutyric acid (GABA) transporter vGAT (*30*).

These data have profound implications with respect to how energy balances across synapses in the brain are achieved and whether different neuronal populations might be more vulnerable than others to presynaptic metabolic compromise due to the total load created by these SV pools. An important aspect of this is understanding how different neurons adjust ATP_presyn_ production to local demands. We previously showed that nerve terminals make use of both feedback and feedforward regulatory mechanisms for ATP_preyn_ production. Feedback mechanisms respond to increased ATP_presyn_ consumption and the ensuing changes in the adenosine 5′-monophosphate (AMP)/ATP ratio (*2*), while feedforward drives ATP_presyn_ production via modulation via a signal that is predictive of upcoming energetic needs, e.g., changes in cytosolic Ca^2+^ (*3*). This feedback pathway would be well suited to adjust ATP_presyn_ levels as basal activity-independent metabolic needs change. Activity-dependent feedforward mechanisms to modulate ATP_presyn_ production such as proposed for the lactate shuttle (*31*) or Ca^2+^-driven mitochondrial regulation (*3*) might not compensate for large resting metabolic loads because they are inherently uncoupled from activity. Thus, depending on what regulatory strategies are available, different neurons may be differentially affected by such high metabolic burdens. Although at face value using a vesicle filling method that results in a continuous ATP_presyn_ consumption appears inefficient, it may simply result from optimizing the speed with which SVs are filled with neurotransmitter. Faster transport is generally achieved by lowering the energy barrier for the key structural transition, which, in turn, implies a higher probability of occurring from thermal fluctuations. Given the vast number of synapses in the human brain and the presence of hundreds of SVs at each these nerve terminals, this hidden metabolic cost of quickly returning synapses in a “ready” state comes at the cost of major ATP_presyn_ and fuel expenditure, likely contributing significantly to the brain’s metabolic demands and metabolic vulnerability.

## METHODS

### Animals

All animal-related experiments were performed in accordance with protocols approved by the Weill Cornell Medicine Institutional Animal Care and Use Committee. Wild- type rats were of the Sprague-Dawley strain (Charles River Laboratories strain code: 400, RRID: RGD_734476).

### Primary neuronal culture

Hippocampal CA1 to CA3 neurons were isolated from 1- to 3-day-old rats of mixed gender and plated on poly-l-ornithine–coated coverslips as previously described (*32*). Calcium phosphate–mediated gene transfer was used to transfect 6- to 8-day-old cultures as described previously (*33*). Neurons were maintained in culture media composed of minimum essential medium (Thermo Fisher Scientific, S1200038), 0.6% glucose, bovine transferrin (0.1 g/liter; Millipore, 616420), insulin (0.25 g/liter), glutamine (0.3 g/liter), 5 to 10% fetal bovine serum (BSA) (Atlanta Biologicals, S11510), 2% B-27 (Thermo Fisher Scientific, 17504-044), and 4 mM cytosine *b*-d-arabinofuranoside. Cultures were incubated at 37°C in a 95% air/5% CO_2_ in a humidified incubator for 14 to 21 days before use.

### Plasmids constructs

The following previously published DNA constructs were used: vGLUT1-pHluorin (*34*), Munc13-1/2 shRNA and Syn-ATP (*1*), vGlut1-shRNA and Syphy-pH (*23*), vMAT2-pH (*28*), cyto-pH (gift of M.E. Hatten), Arclight (*35*), and TeNT-LC (gift of M. Dong). DNA constructs made for this project are as follows: Human vGlut1 shRNA-resistant rescue and vMAT2.

### Live-cell imaging

Live-cell imaging was performed using a custom-built laser-illuminated epifluorescence microscope with an Andor iXon+ (model no. DU-897E-BV) back-illuminated electron-multiplying charge-coupled device camera that was selected for low dark currents. Transistor–transistor logic (TTL)-controlled Coherent OBIS 488- and 561-nm lasers were used for illumination. Images were acquired through a 40× 1.3 numerical aperture (NA) Fluar Zeiss objective. Experiments were performed at a clamped temperature of 37°C (or 25°C; fig. S2) using a custom-built objective heater under feedback control. Action potentials were evoked by passing 1-ms current pulses, yielding fields of approximately 10 V cm^−1^ via platinum-iridium electrodes. Neurons were continuously perfused at 0.1 ml min^-1^ with a Tyrode’s solution containing 119 mM NaCl, 2.5 mM KCl, 2 mM CaCl2, 2 mM MgCl2, 30 mM glucose, 0.01 mM 6-cyano-7-nitroquinoxaline-2,3-dione, 0.05 mM d,l-2-amino-5-phospho-novaleric acid, and 0.3 μM TTX, buffered to pH 7.4 using 25 mM Hepes. Tyrode’s solution without TTX was used for electrical stimulation experiments. Bafilomycin solutions were prepared freshly from a 250 μM stock into a Tyrode’s + TTX solution and administrated as indicated in each experiment. In experiments without glucose, Tyrode’s molarity was compensated with either 2DG or Hepes (0-glucose). Hypertonic solutions were freshly prepared adding 500 mM sucrose into a Tyrode’s + TTX solution.

### ATP measurements

Luminescence imaging of the ATP_presyn_ reporter, Syn-ATP, was performed as previously described (*1*), with the following modifications: (i) All data acquisition and microscope control were carried out remotely, allowing the microscope to be kept in complete light isolation; (ii) all images were acquired through a 40× 1.3 NA Fluar Zeiss objective using an ET570LP emission dichroic filter (Chroma) (for fluorescence and luminescence); (iii) mCherry fluorescence was excited using a 561-nm OBIS laser (Coherent) that was gated to be on only during mCherry image acquisition to avoid creating spurious background light during the luminescence image acquisition; (iv) long time series of imaging pairs (fluorescence and luminescence) was automated using a custom-written Andor Basic program; and (v) given that we did not observe significant differences in pH changes (fig. S5) when comparing wild-type neurons in control, in bafilomycin, and vGlut1-KD neurons, we did not correct ATP measurements for changes in cytosolic pH. On the basis of the results reported previously (*1*), we assumed a linear correlation between the L/F and ATP concentration. Therefore, ATP_presyn_ is reported as normalized values in each case to the starting L/F.

Ouabain [1 mM] and bafilomycin [1 μM] experiments were done separately with their own paired controls. ATP_presyn_ consumption rate was calculated as the slope of a line fit on L/F control traces between 1- and 6-min 2DG interval. Our measurement region of interest at each nerve terminal was a circular region of radius ~1 μm, corresponding to a volume of ~4.2 μm^3^. We previously showed that the average resting ATP_presyn_ concentration is ~1.4 mM, which converts to 3.5 × 10^6^ ATP molecules in our measurement volume. The initial rate of decay during 2DG perfusion was 5.4%/min (Fig. 1B) corresponding to an initial ATP consumption rate of ~3100 ATP_presyn_ molecules/s.

### pHluorin measurements

pHluorin signals during electrical stimulation are reported as a percentage of the total vesicle pool, whose fluorescence is obtained by perfusion of a Tyrode’s solution containing 50 mM NH_4_Cl buffered at pH 7.4 using 25 mM Hepes and denoted as Δ*F*_NH4Cl_ (*33*). pHluorin signals in resting neurons are reported as a percentage of the cytosolic pH 6.9, obtained as follows

$$\%F_{6.9\text{pH}}=(\Delta F_{\text{baf}}/\Delta F_{\text{NH}4\text{Cl}})*\alpha$$ (1)

where α is the correction factor to convert fluorescence determined at pH 7.4 (our NH_4_Cl pH value) to that expected for the pH in the cytosol (pH 6.9) calculated as

$$\alpha =(1-10^{\left(\text{pK}-6.9\right)})/(1-10^{\left(\text{pK}-7.4\right)})\approx 1.72$$ (2)

pHluorin *pK* is 7.1 (*36*).

Images from pHluorin signals in resting neurons were acquired in Tyrode’s + TTX solution for at least 2 min, followed by 500 nM bafilomycin perfusion for at least 15 min. For experiments inhibiting the vMAT2 transporter, 100 nM reserpine was perfused during the entire experiment (with Tyrode’s + TTX and with bafilomycin Tyrode’s + TTX).

In experiments using Syphy-pH, glutamatergic neurons were identified by loading Oyster 550–labeled rabbit anti-vGAT (Synaptic Systems, catalog no. 131-103C3), as previously described (*25*). Using our standard promoters for reporter expression (cytomegalovirus), in a pilot study with more than 20 dishes with several cells per dish transfected, we found that none of the transfected cells were actually GABAergic (Oyster-positive–labeled). Therefore, non-Oyster–labeled boutons were assumed to be glutamatergic neurons.

### Optical membrane potential measurements

Synaptic membrane potential changes were measured by using the genetically encoded plasma membrane–localized voltage sensor, Arclight (*35*). Images from Arclight signals in resting neuronal presynaptic boutons were acquired in Tyrode’s solution without TTX for at least 2.5 min before perfusion of 1 mM ouabain for at least 6 min. Voltage signals are reported as percentage of maximal membrane depolarization change, whose fluorescence is obtained by perfusion of a Tyrode’s solution containing 80 mM KCl, which rapidly depolarizes the membrane and reaches an stable plateau (fig. S1Aa).

### Cytosolic Ca^2+^ measurements

Cytosolic free Ca^2+^ changes were measured by using GCaMP6f sensor. Images from GCaMP6f signals in resting neuronal presynaptic boutons were acquired in Tyrode’s solution with or without TTX for at least 5 min before perfusion of 1 mM ouabain. All changes in fluorescence were normalized to GCaMP6f intensity to Ca^2+^ saturation, which was calculated by applying a solution of Tyrode’s buffer (pH 6.9) at the end of experiments containing ionomycin (1 mM).

### Immunocytochemistry and antibodies

Neurons were fixed with 4% paraformaldehyde, permeablized with 0.25% Triton X-100, and blocked for 10 min at room temperature with 5% BSA. Primary antibodies were diluted with 5% BSA and incubated with the cells at room temperature for 1 hour. After 3× 5-min washes in phosphate-buffered saline (PBS), cells where incubated with secondary antibodies, followed by additional 3× 5-min washes in PBS. Guinea pig anti-vGlut1 polyclonal antibody (Millipore, AB1905) was used at 1:1000, followed by goat anti–guinea pig Alexa Fluor 546. FluoTag-X2 anti-TagFp atto 488 (Nanotag, N0502) was used at 1:500. Immunofluorescence images were taken by the camera in a similar way as live-cell imaging.

### Image analysis and statistics

Images were analyzed using the ImageJ plug-in Time Series Analyzer V3 where 20 to 30 circular regions of interest (ROIs) of radius ~1 μm corresponding to synaptic boutons expressing the pHluorin (as determined with NH_4_Cl superfusion) or SynATP (mCherry positive) were selected, and the fluorescence was measured over time. Image loading and posterior raw data saving were automatized using a homemade Python code for Fiji. ROIs signals were analyzed using homemade script routines in Igor-pro v6.3.7.2 (Wavemetric, Lake Oswego, OR, USA). Proton efflux rate was calculated as the slope of a line fit on pHluorin traces between 50- and 400-s bafilomycin interval. Results of group data analysis are presented as means ± SEM. When analyzing means, *P* values are based on the nonparametric Wilcoxon-Mann-Whitney test. *P* < 0.05 was considered significant and denoted with a single asterisk, whereas *P* < 0.01, *P* < 0.001, and *P* < 0.0001 are denoted with two, three, and four asterisks, respectively. The *n* value, indicated in the figure legends for each experiment, represents the number of cells imaged.

## Notes

View/request a protocol for this paper from *Bio-protocol*.
