# Supplementary material for: Synaptic vesicle pools are a major hidden resting metabolic burden of nerve terminals
Source: Sci Adv. 2021 Dec 3;7(49):eabi9027. doi: 10.1126/sciadv.abi9027 (PMC8641928; doi:10.1126/sciadv.abi9027)
Supplement: Supplementary file 1 — Figs. S1 to S6 [file sciadv.abi9027_sm.pdf]

Science Advances
AAAS

Supplementary Materials for

Synaptic vesicle pools are a major hidden resting metabolic burden of nerve terminals

Camila Pulido and Timothy A. Ryan*

*Corresponding author. Email: taryan@med.cornell.edu

Published 3 December 2021, *Sci. Adv.* **7**, eabi9027 (2021)
DOI: 10.1126/sciadv.abi9027

This PDF file includes:

Figs. S1 to S6

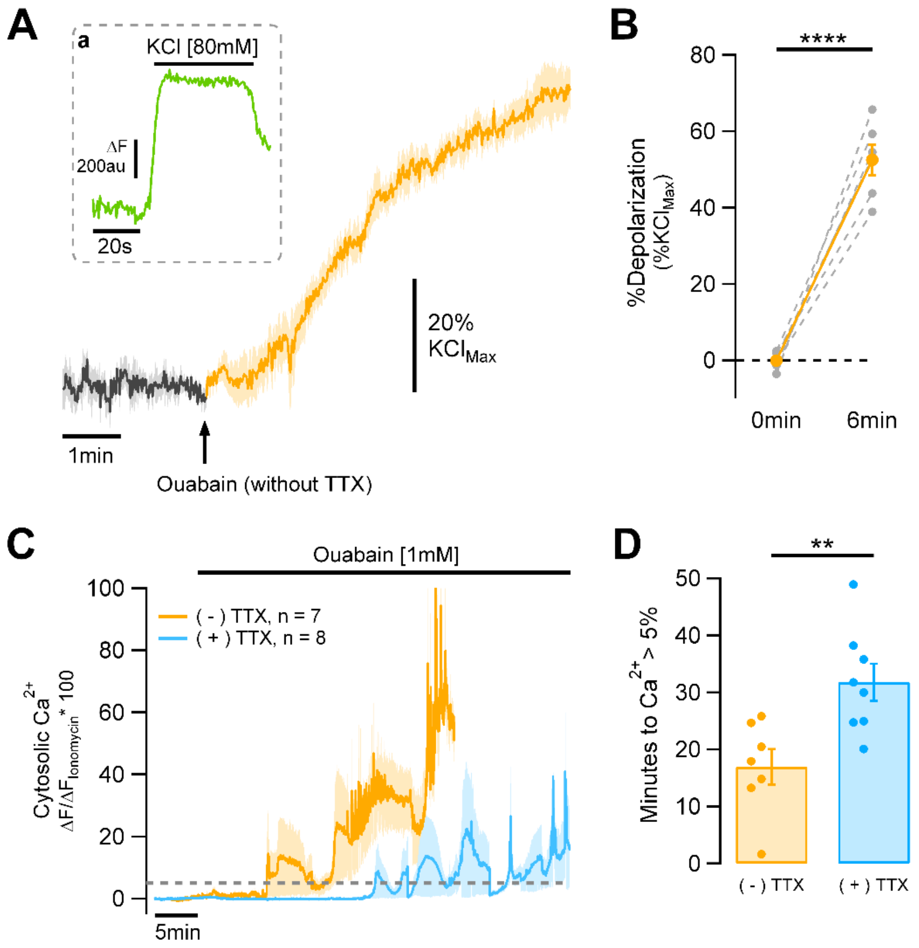

Fig S1. Inhibition of Na^+/K^+ pump by ouabain causes a rapid membrane depolarization but not an immediate raise in cytosolic Ca^{2+} at nerve terminals. A and **B** Membrane potential measured in the absence of TTX from synaptic boutons expressing Arclight. **A**, Average Arclight trace of WT neurons before (black trace) and after addition of 1mM ouabain (orange trace). Inverted fluorescence expressed as a percentage of the maximal (inverted) fluorescence change on perfusion with 80mM KCl per neuron. **a,** Representative Arclight trace showing fluorescent plateau upon application of 80mM KCl. **B,** Ouabain causes a rapid fluorescence change associated with depolarization of the membrane potential (0min = -0.1%KCl_{Max} ± 0.9 vs 6min: 52.51%KCl_{Max} ± 4.03; n = 6, ****p < 0.0001). **C,** Average GCaMP6f trace of WT neurons in presence (orange trace, n = 7) or absence of TTX (blue trace, n = 8) before and after perfusion with 1mM ouabain. Fluorescence expressed as a percentage of the maximal ΔF from Ionomycin treatment. ± SEM intervals are indicated by shaded colored areas. Dashed gray line indicates 5% level. **D,** Ouabain application does not cause an immediate raise in the

cytosolic free Ca^{2+}. Ouabain is applied for several minutes before cytosolic Ca^{2+} levels exceed 5% of the maximal ΔF from Ionomycin treatment in neurons treated with TTX (blue dots) or without TTX (orange dots), respectively as: 31.8min ± 3.24 and 17min ± 3.1. Error bars indicate SEM. **$p < 0.01$, Wilcoxon-Mann-Whitney test.

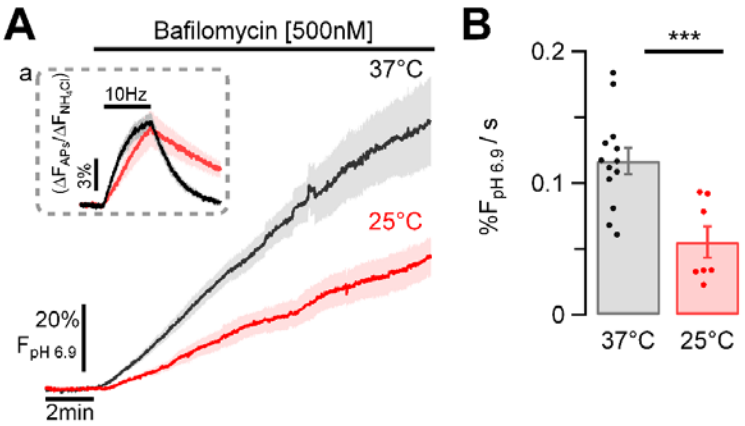

Fig S2. **H⁺ efflux is temperature dependent.** H^+ flux from synaptic vesicles measured in hippocampal neurons expressing vG-pH. **A,** Average vG-pH traces of control neurons at either 37°C or at 25°C in presence of TTX before and after perfusion with bafilomycin. Fluorescence expressed as a percentage of the maximum fluorescence change expected for pH = 6.9 (%$F_{pH6.9}$) based on perfusion with NH_4Cl (see methods) ± SEM intervals are indicated by shaded colored areas. **a,** Average vG-pH traces in response to 100APs. **B,** Lowering the temperature from 37°C to 25°C reduced the SV H^+ efflux rate by a factor of ~2. (37°C: 0.117% $F_{pH6.9}$/s ± 0.01 (n = 13) vs 25°C: 0.055% $Fp_{H6.9}$/s ± 0.012 (n = 7)). Error bars indicate SEM. ***p < 0.001, Wilcoxon-Mann-Whitney test.

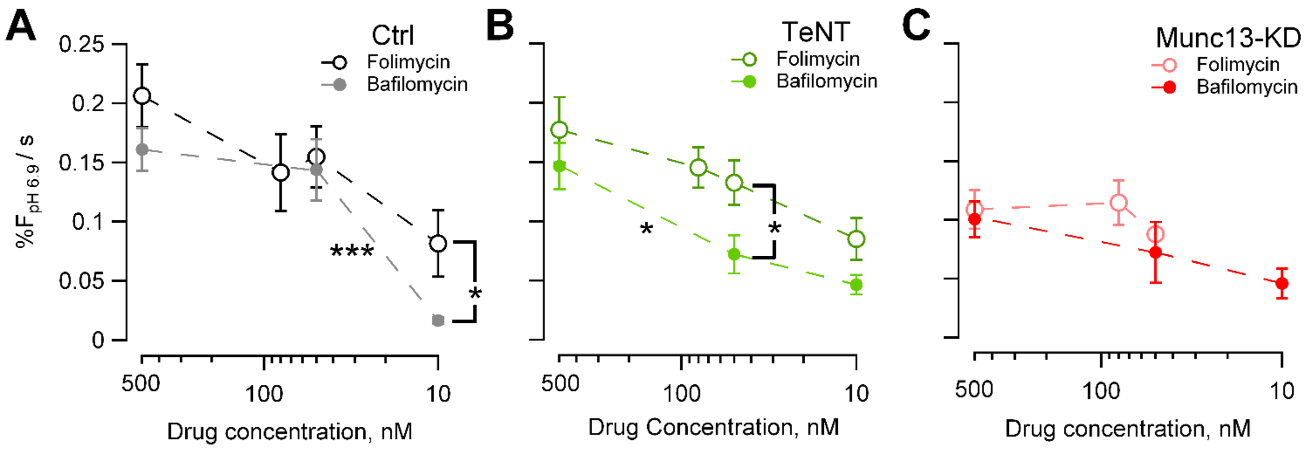

Fig S3. Unmasking SV H+ efflux depends on ATPase block. SV H^+ efflux from synaptic vesicles expressing vG-pH measured in the absence of TTX and at different concentration of bafilomycin (close symbols) or folimycin (open symbols). Concentration ranged from 10nM – 500nM. **A,** Control neurons. In bafilomycin control (gray line, close symbols), H^+ flux change from 50nM to 500nM was not significant different (50nM: 0.14%$F_{pH6.9}$/s ± 0.025 (n = 9) vs 500nM: 0.16%$F_{pH6.9}$/s ± 0.017 (n = 11); n.s. p = 0.6), therefore, we used 500nM as the effective V-ATPase blockage to measure SV H^+ efflux. **B** and **C,** Neurons where exocytosis was genetically suppressed. **B,** Neurons expressing tetanus toxin light chain (TeNT). **C,** Neurons suppressing expression of Munc13 (Munc13-KD). Error bars indicate SEM. *p < 0.05, ***p < 0.001, Wilcoxon-Mann-Whitney test.

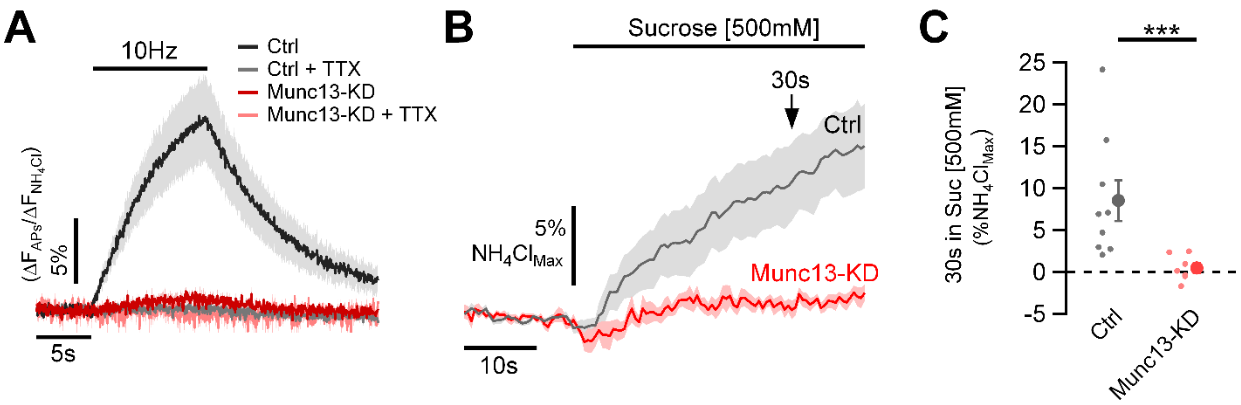

Fig S4. **Spontaneous synaptic vesicle exocytosis is suppressed in Munc13-KD neurons**. Spontaneous release measured from synaptic vesicles expressing vG-pH upon addition of a hypertonic solution (Sucrose [500mM]). **A,** Average vG-pH traces in response to 100APs (10Hz) in control neurons (Ctrl, black trace, n = 9) or in neurons where exocytosis is genetically suppressed by ablating expression of Munc13 (Munc13-KD, red trace, n = 7), before (dark color tone) and after addition of TTX (light color tone). ΔF values are normalized to maximal ΔF from NH4Cl treatment (ΔF/ΔFNH_4Cl). **B,** vG-pH average traces of Ctrl and Munc13-KD neurons measured in presence of TTX before and after perfusion with Sucrose [500mM]. Fluorescence expressed as a percentage of the maximal ΔF from NH_4Cl treatment. ± SEM intervals are indicated by shaded colored areas. **C,** Hypertonic sucrose application increases the rate of spontaneous exocytosis in control neurons as measured with vG-pH fluorescence (normalized to ΔFNH_4Cl) but not in Munc13-KD neurons. Percentage of fluorescent increase in Ctrl and Munc13-KD neurons measured 30s after application of sucrose, respectively as: 8.54%NH_4Cl_{Max} ± 2.4 and 0.48%NH_4Cl_{Max} ± 0.58. Error bars indicate SEM. ***$p < 0.001$, Wilcoxon-Mann-Whitney test.

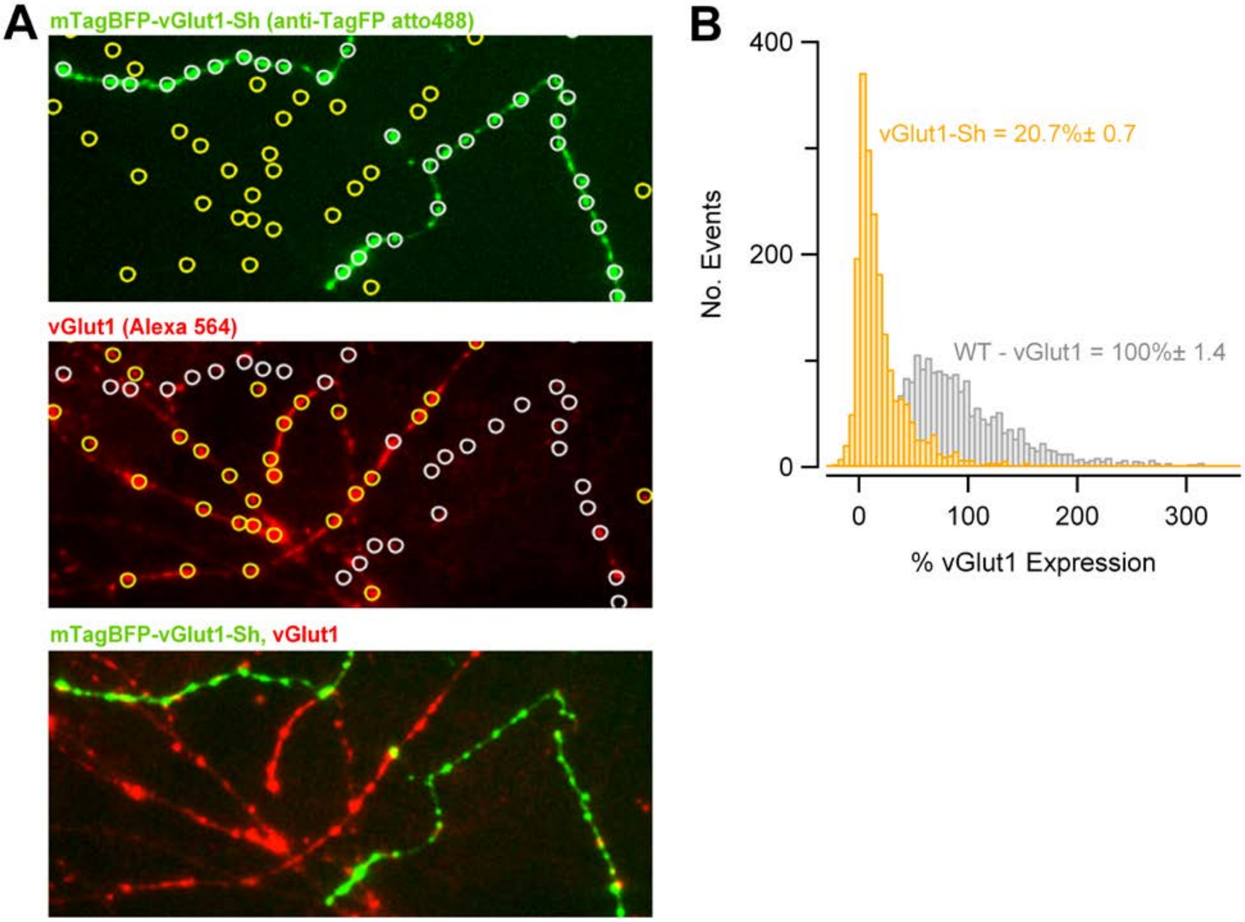

Fig S5. Expression of a ShRNA targeting vGlut1 resulted in ~80% suppression of vGlut1 expression. A, Representative presynaptic boutons from a neuron expressing an mTagBFP-vGlut1-Sh (stained with FlutoTag-X2 anti-TagFP Atto488). BFP-positive boutons (white circles) show much lower vGlut1 immunofluorescence when compared with neighboring non-transfected boutons (yellow circles). **B** Distribution of vGlut1 expression in boutons transfected with mTagBFP-vGlut1-Sh (n = 2050) normalized to their respective neighboring non-transfected boutons (n = 1904) from 24 neurons. Respectively as: mean ± SEM: 20.7%± 0.7 vs 100% ± 1.4.

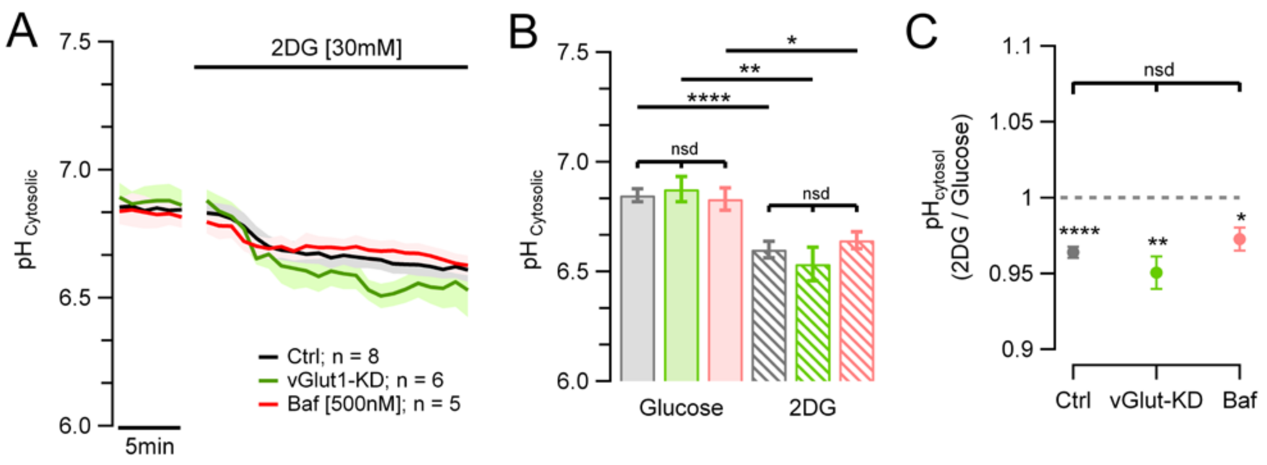

Fig S6. Changes in cytosolic pH during 2DG perfusion is similar in vGlut1-KD neurons or during bafilomycin treatment. Cytosolic pH measurements from hippocampal neurons expressing a cytoplasmic pHluorin. **A,** Average cytosolic pH traces in WT neurons in control (n = 8), in Bafilomycin (n = 5) and vGlut1-KD (n = 6) in presence of glucose (first 5min) and 2DG. **B,** 2DG causes a cytosolic pH decrease, as previously reported (*1*). Ctrl: $pH_{Glucose}$ 6.85 ± 0.03 vs pH_{2DG} 6.6 ± 0.04 (n = 8; $p < 0.0001$); bafilomycin: $pH_{Glucose}$ 6.83 ± 0.04 vs pH_{2DG} 6.64 ± 0.04 (n = 5; $p < 0.02$); vGlut1-KD: $pH_{Glucose}$ 6.87± 0.04 vs pH_{2DG} 6.53 ± 0.08 (n = 6; $p < 0.005$). **C,** Similar pH decrease was observed in control, vGlut1-KD and bafilomycin neurons.
